# Novel Bioluminescent Binding Assays for Ligand–Receptor Interaction Studies of the Fibroblast Growth Factor Family

**DOI:** 10.1371/journal.pone.0159263

**Published:** 2016-07-14

**Authors:** Ge Song, Xiao-Xia Shao, Qing-Ping Wu, Zeng-Guang Xu, Ya-Li Liu, Zhan-Yun Guo

**Affiliations:** Research Center for Translational Medicine at East Hospital, College of Life Sciences and Technology, Tongji University, Shanghai, China; Institute of Biomedicine, FINLAND

## Abstract

We recently developed novel bioluminescent binding assays for several protein/peptide hormones to study their interactions with receptors using the so far brightest NanoLuc reporter. To validate the novel bioluminescent binding assay using a variety of protein/peptide hormones, in the present work we applied it to the fibroblast growth factor (FGF) family using the prototype member FGF2 as an example. A fully active recombinant FGF2 retaining a unique exposed cysteine (Cys) residue was chemically conjugated with an engineered NanoLuc carrying a unique exposed Cys residue at the C-terminus via formation of an intermolecular disulfide linkage. The NanoLuc-conjugated FGF2 (FGF2-Luc) retained high binding affinity to the overexpressed FGFR1 and the endogenous FGF receptor with the calculated dissociation constants of 161 ± 21 pM (*n* = 3) and 25 ± 4 pM (*n* = 3), respectively. In competition binding assays using FGF2-Luc as a tracer, receptor-binding potencies of wild-type or mutant FGF2s were accurately quantified. Thus, FGF2-Luc represents a novel non-radioactive tracer for the quantitative measurement of ligand–receptor interactions in the FGF family. These data suggest that the novel bioluminescent binding assay can be applied to a variety of protein/peptide hormones for ligand–receptor interaction studies.

## Introduction

Protein/peptide hormones include all secretory proteins and peptides with signal transduction functions. They are the largest group of endogenous signaling molecules and play a variety of biological functions mediated by specific cell membrane receptors. To study their interactions with receptors, ligand–receptor binding assays have been used for decades. However, conventional binding assays rely on radioligands that have drawbacks, such as radioactive hazards and short shelf lives, and can only be used in laboratories with a radioactive material license. In recent studies [1–5], we developed novel bioluminescent binding assays for several protein/peptide hormones using nanoluciferase (NanoLuc) as a reporter, because it has the brightest bioluminescence reported to date [6]. The NanoLuc reporter was either chemically conjugated at an appropriate position or genetically fused at one terminus of the target protein/peptide, and the resultant NanoLuc-conjugated/fused protein/peptide was a novel bioluminescent ligand, provided it retained high receptor-binding affinity. The novel bioluminescent ligands represent a novel class of non-radioactive tracers for quantitative measurements of ligand–receptor interactions [7]. However, the novel bioluminescent binding assay needs to be validated using a variety of protein/peptide hormones due to the high diversity of protein/peptide hormones and their receptors.

Fibroblast growth factors (FGFs) are a group of small proteins that share similar structural characteristics and have multiple functions, such as angiogenesis, wound healing, and embryonic development [8–11]. In humans, 22 FGFs have been identified, which play a variety of functions in paracrine, endocrine, or intracrine manners. The paracrine and endocrine FGFs bind and activate four transmembrane FGF receptors (FGFR1–4), which have three extracellular immunoglobulin-like domains and a split intracellular tyrosine kinase domain [12–14]. Paracrine FGFs bind extracellular heparan sulfate as a cofactor, whereas endocrine FGFs use the transmembrane protein α-Klotho or β-Klotho as a cofactor [12–14].

FGF2 (also known as basic FGF) is a prototype of the FGF family and was first isolated together with FGF1 (also known as acidic FGF) in the early 1970s [15,16]. In the 1980s, its gene was cloned, which encodes five different protein isoforms due to alternative translation initiation from upstream CUG codons [17–19]. The CUG-initiated FGF2 isoforms are localized in the nucleus, and the AUG-initiated form is mostly in the cytosol because it does not contain the classical signal peptide sequence for efficient secretion. Secretion of AUG-initiated FGF2 is mediated by an unconventional pathway with lower efficiency [20]. FGF2 folds into a globular structure [21,22] and binds with high affinity to the IIIb and IIIc variants of FGFR1, the IIIc variant of FGFR2, the IIIc variant of FGFR3, and FGFR4. The mechanism underlying its interaction with receptors has been extensively studied, and some crystal structures of FGF2 in complex with its receptors have been solved [23–25].

In previous receptor-binding assays, radioactive iodine-125 (^125^I)-labeled FGF2 was used as a radioligand [26–32]. In this study, we applied the newly developed bioluminescent binding assay to FGF2 for interaction studies with its receptors. The results suggest that the novel bioluminescent ligand–receptor binding assay can be applied to a wide range of protein/peptide hormones.

## Materials and Methods

### Generation of expression constructs for wild-type or mutant FGF2s

The nucleotide sequence encoding AUG-initiated human FGF2 (154 amino acids without the first Met) was chemically synthesized. After cleavage with the restriction enzymes NdeI and EcoRI, the synthetic DNA fragment was ligated into a pET expression vector pretreated with the same restriction enzymes. The resultant expression construct, pET/6×His-FGF2, encoded a human FGF2 carrying an N-terminal 6×His-tag and an enterokinase cleavage site (amino acid sequence MHHHHHHMDDDDK). To generate FGF2 mutants carrying a single mutation (C95S, Y32A, or Y111A), site-directed mutagenesis was performed using the construct pET/6×His-FGF2 as a template. The nucleotide sequences of the wild-type or mutant FGF2s were confirmed by DNA sequencing.

### Overexpression and purification of wild-type or mutant FGF2s

The expression constructs for wild-type or mutant FGF2s were transformed into the *Escherichia coli* strain BL21(DE3)star, respectively. The transformants were cultured in liquid LB medium containing 100 mg/l ampicillin at 37°C to OD600 ~1.0 with vigorous shaking (250 rpm) in a SPH-211C shaker (Shiping Temperature, Shanghai, China). Thereafter, isopropyl β-D-1-thioglactopyranoside (IPTG) stock solution was added to a final concentration of 1.0 mM, and the *E*. *coli* cells were continuously cultured at 25°C overnight with gentle shaking (150 rpm). After induction, the *E*. *coli* cells were collected by centrifugation (5,000 *g*, 10 min), resuspended in the lysis buffer (20 mM phosphate, 0.5 M NaCl, 50 mM dithiothreitol, pH7.5), and lysed by sonication. Subsequently, the lysate was subjected to centrifugation (10,000 *g*, 30 min) and the supernatant was loaded onto a heparin-Sepharose affinity column. The bound wild-type or mutant FGF2 was eluted from the affinity column with high salt solution (20 mM phosphate, 2.5 M NaCl, pH7.5) and manually collected. The eluted FGF2 fraction was analyzed by sodium dodecyl sulfate-polyacrylamide gel electrophoresis (SDS-PAGE) and quantified with the 2,2’-bicinchonic acid (BCA) method using bovine serum albumin (BSA) as a standard.

### Receptor activation assays of wild-type or mutant FGF2s

The biological activities of recombinant FGF2s were assayed as receptor activation potency using a serum response element (SRE)-controlled NanoLuc reporter. Briefly, human embryonic kidney (HEK) 293T cells were transfected the reporter vector pNL1.2/SRE using the transfection reagent Lipofectamine 2000 (Invitrogen, Carlsbad, CA, USA). The next day, the transfected cells were trypsinized, seeded into a 96-well plate, and cultured in complete medium (DMEM medium supplemented with 10% fetal bovine serum, 100 U/ml penicillin and 100 μg/ml streptomycin) at 37°C overnight to ~80% confluence. Thereafter, the complete medium was removed and the cells were continuously cultured in serum-free DMEM medium at 37°C for 12 h. Subsequently, the serum-free medium was removed, the assay solution (serum-free DMEM medium plus 1% BSA) containing various concentrations of wild-type or mutant FGF2 was added (100 μl/well), and the cells were continuously cultured at 37°C for 4 h. Thereafter, the assay solution was removed and the cells were lysed with lysis solution (100 μl/well, from Promega, Madison, WI, USA). Then, the cell lysate was transferred to a white opaque 96-well plate (50 μl/well) and bioluminescence was immediately measured on a Spectramax M5 plate reader (Molecular Devices, Sunnyvale, CA, USA) after addition of the diluted substrate (50 μl/well) using a 8-channel pipette. The measured bioluminescence data were expressed as mean ± standard error (SE) (*n* = 3) and fitted to sigmoidal curves using the SigmaPlot 10.0 software.

### Chemical conjugation of 6×His-[C95S]FGF2 with an engineered NanoLuc reporter

The engineered 6×His-NanoLuc-Cys, which carries a unique exposed cysteine (Cys) residue at the C-terminus, was overexpressed in *E*. *coli* and activated by 2,2’-dipyridyl disulfide according to our previous procedure [3]. For conjugation with the activated 6×His-NanoLuc-Cys, ~50 μg of the purified 6×His-[C95S]FGF2, eluted from the heparin affinity column, was diluted 5-fold with 50 mM phosphate buffer (pH7.5), and then added to a small spin filtration column containing ~50 μl heparin-Sepharose resin. After being washed with washing buffer (20 mM phosphate, 0.5 M NaCl, pH7.5), ~120 μg of the activated 6×His-NanoLuc-Cys (in 20 mM phosphate buffer with ~0.5 M NaCl) was added to the resin. After incubation at 25°C for 30 min, the resin was washed with washing buffer and eluted with 150 μl elution buffer [phosphate buffered saline (PBS) plus 50 mg/ml heparin). The eluted conjugate fraction was analyzed by bioluminescence measurement and non-reducing SDS-PAGE, and stored at -80°C after addition of 10% of glycerol.

### Receptor-binding assays using the overexpressed receptor

HEK293T cells were transiently transfected with the human FGFR1 (transcript variant 2, NCBI reference sequence: NM_015850) expression construct pENTER/FGFR1 (Vigene Bioscience, Rockville, MD, USA) using the Lipofectamine 2000 transfection reagent (Invitrogen). The next day, the transfected cells were trypsinized, seeded into a 96-well plate, and cultured in complete medium to ~90% confluence. To start the binding assay, medium was removed and the binding solution (serum-free DMEM medium plus 1% BSA and 5 μg/ml heparin) was added (100 μl/well). For the saturation binding assay, the assay solution contained various concentrations of the NanoLuc-conjugated FGF2 (FGF2-Luc). The nonspecific binding data were obtained by competition with 250 nM 6×His-FGF2. For the competition binding assay, the binding solution contained a constant concentration of FGF2-Luc and various concentrations of wild-type or mutant FGF2. After binding at 20°C for 2–3 h, the binding solution was removed and the cells were washed twice with ice-cold PBS (200 μl/well for each wash). Finally, the cells were lysed with lysis solution (100 μl/well, from Promega), and the cell lysate was transferred to a white opaque 96-well plate (50 μl/well). Bioluminescence was immediately measured on a Spectramax M5 plate reader (Molecular Devices) after addition of the diluted substrate (50 μl/well) using an 8-channel pipette. The measured bioluminescence data were expressed as mean ± SE (*n* = 3) and fitted to a one-site binding model using the SigmaPlot 10.0 software.

### Receptor-binding assays using the endogenous receptor

The untransfected HEK293T cells were detached using 1.0 mM ethylenediaminetetraacetic acid (in PBS), thoroughly washed with DMEM medium, and resuspended in the binding solution (serum-free DMEM medium plus 1% BSA and 5 μg/ml heparin) at a density of 10^6^ cells/ml. Thereafter, the cell suspension was added to a 96-well filtration plate (100 μl/well), and mixed with an equal volume of binding solution either containing various concentrations of FGF2-Luc (saturation binding assay) or containing a constant concentration of FGF2-Luc and various concentrations of wild-type or mutant FGF2 (competition binding assay). After binding at 20°C for 2–3 h, the binding solution was removed by centrifugation (400 *g*, 2 min) and the cells were washed twice with ice-cold PBS (200 μl/well for each wash) by centrifugation (400 *g*, 2 min). Finally, the cells were resuspended in PBS (100 μl/well) and transferred to a white opaque 96-well plate (50 μl/well). After addition of the lysis solution (25 μl/well) and the diluted substrate (25 μl/well) using an 8-channel pipette, bioluminescence was immediately measured on a Spectramax M5 plate reader (Molecular Devices). The measured bioluminescence data were expressed as mean ± SE (*n* = 3) and fitted to a one-site binding model using the SigmaPlot 10.0 software.

## Results and Discussion

### Strategy for site-specific conjugation of FGF2 with the NanoLuc reporter

**Fig 1:**
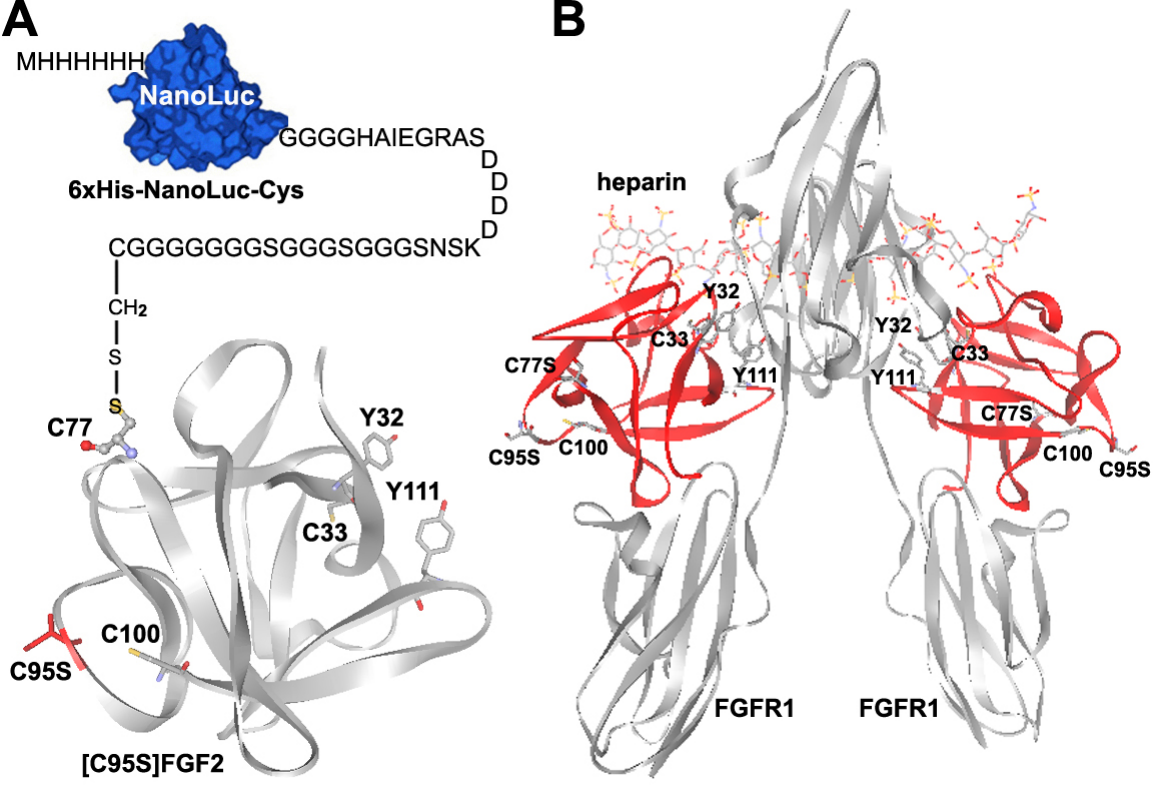
**(A)** Schematic presentation of the NanoLuc-conjugated FGF2. The structure of [C95S]FGF2 was adopted from the previously published FGF2 crystal structure (PBD ID: 2BFH) [22]. Positions of the Cys residues, Y32, and Y111 in FGF2 are labeled and their side-chains are shown. **(B)** The previously solved crystal of structure of FGF2 in complex with receptor FGFR1 and heparin (PBD ID: 1FQ9) [24]. FGF2 molecules are shown in red and the positions of their Cys residues, Y32, and Y111 are labeled.

To apply the novel bioluminescent ligand–receptor binding assay to FGF2, the NanoLuc reporter needs to be covalently linked to an appropriate position of FGF2 using a practical approach. As shown in Fig 1A, FGF2 folds into a globular structure with four free Cys residues: C33 and C100 are buried in the interior, whereas C77 and C95 are exposed at the surface [21,22]. As shown in Fig 1B, positions of the exposed Cys residues (replaced with serine to prevent aggregation in this structure) of FGF2 are located at the opposite side of the receptor-binding and heparin-binding sites [23–25]. Thus, the exposed Cys residues are suitable sites for chemical conjugation with our previously designed 6×His-NanoLuc-Cys [3], which carries a unique exposed Cys residue at the C-terminus for conjugation with various proteins or peptides. To obtain homogenous NanoLuc-conjugated FGF2, we replaced the exposed C95 residue with a serine; thus, the resultant [C95S]FGF2 retained a unique exposed Cys residue (C77) and could be site-specifically conjugated with the engineered NanoLuc reporter using our previous procedure [3].

### Overexpression, purification, and characterization of wild-type or mutant FGF2s

**Fig 2:**
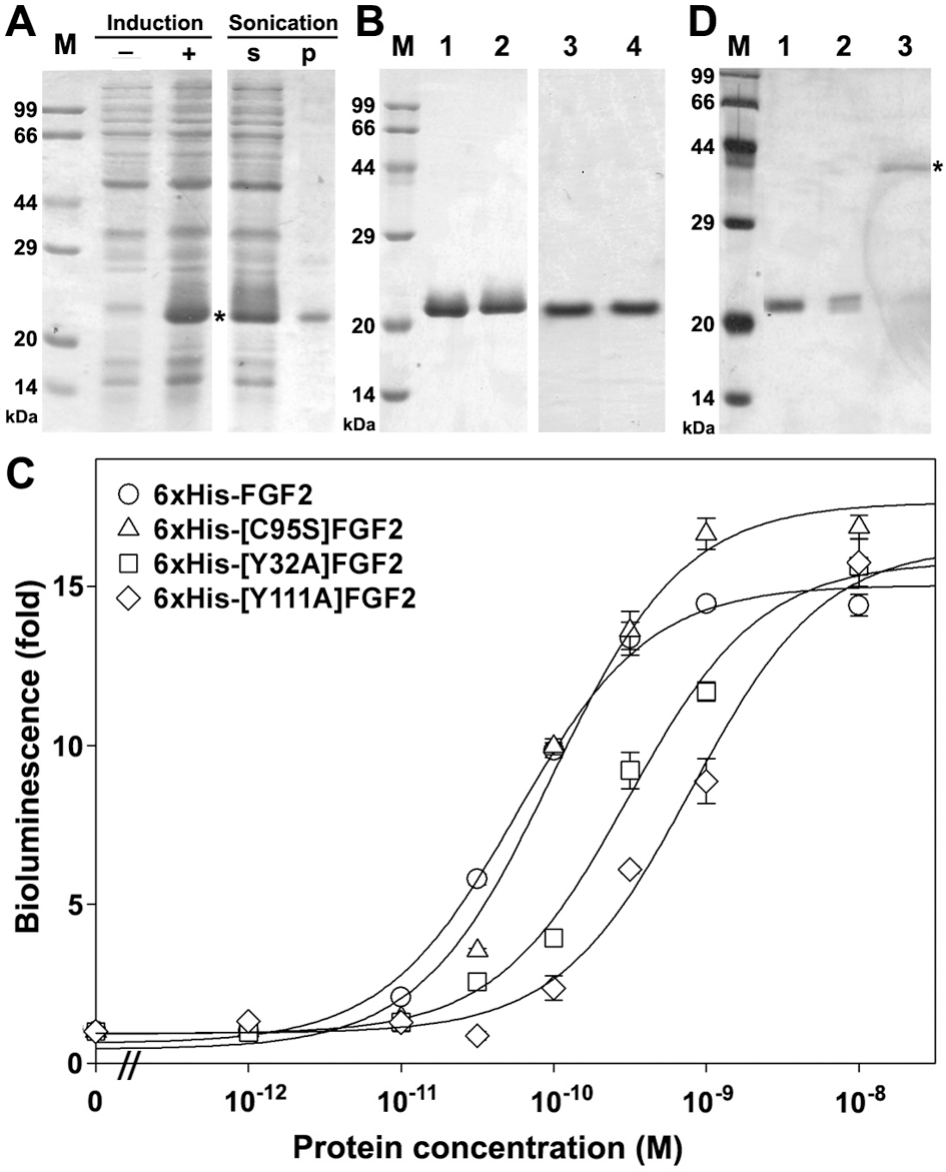
**(A)** SDS-PAGE analysis of 6×His-FGF2 expression in *E*. *coli*. Lane (-), before IPTG induction; lane (+), after overnight IPTG induction; lane (s), supernatant of the cell lysate after sonication; lane (p), pellet of the cell lysate after sonication. The loading amount in each lane was equal to the *E*. *coli* cells from 50 μl of the culture broth. After electrophoresis, the gel was stained with Coomaissie Brilliant Blue R250. The band representing 6×His-FGF2 is indicated by an asterisk. **(B)** SDS-PAGE analysis of the purified wild-type or mutant FGF2s. After elution from a heparin-Sepharose column, 1 μg of the purified protein was loaded onto the non-reducing SDS-gel. Lane 1, 6×His-FGF2; lane 2, 6×His-[C95S]FGF2; lane 3, 6×His-[Y32A]FGF2; lane 4, 6×His-[Y111A]FGF2. After electrophoresis, the gel was stained with Coomaissie Brilliant Blue R250. **(C)** Receptor activation assays of the purified wild-type or mutant FGF2s. HEK293T cells were transfected with the SRE-controlled NanoLuc reporter vector. After being serum starved for 12 h, the transfected cells were treated with the wild-type or mutant FGF2s for 4 h and then lysed for bioluminescence measurement. The measured data were expressed as mean ± SE (*n* = 3) and fitted to sigmoidal curves using the SigmaPlot10.0 software. **(D)** SDS-PAGE analysis of the FGF2-Luc conjugate. Lane 1, 6×His-[C95S]FGF2 used for conjugation; lane 2, the washed fraction; lane 3, the eluted fraction. Samples were loaded onto a non-reducing SDS-gel and the gel was silver stained after electrophoresis. The band representing FGF2-Luc is indicated by an asterisk.

To overexpress FGF2 in *E*. *coli*, the coding nucleotide sequence of human FGF2 was chemically synthesized using the *E*. *coli*-optimized codons to increase its expression level. To facilitate purification, a 6×His-tag was present at the N-terminus of the recombinant FGF2. To remove the 6×His-tag after purification, an enterokinase cleavage site (DDDDK) was introduced between the tag and the mature FGF2. The nucleotide sequence and amino acid sequence of the resultant 6×His-FGF2 are shown in the supplementary S1 Fig. For site-specific conjugation with the NanoLuc reporter, we generated 6×His-[C95S]FGF2 that carries a unique exposed Cys residue (C77) as the conjugation site. To test whether the NanoLuc-conjugated FGF2 could discriminate receptor-binding potencies of different ligands, we generated 6×His-[Y32A]FGF2 and 6×His-[Y111A]FGF2. As shown in Fig 1B, both Tyr32 and Tyr111 interact with FGF receptor [23–25], thus we deduced that their mutation would decrease the receptor-binding potency of FGF2. The wild-type or mutant FGF2s were efficiently overexpressed in *E*. *coli* as soluble proteins (Fig 2A), and were purified to homogeneity using single-step heparin-Sepharose affinity chromatography, as analyzed by SDS-PAGE (Fig 2B). From 1 L of the *E*. *coli* culture broth, typically ~80 mg of purified wild-type or mutant FGF2s could be obtained; thus, these proteins could be conveniently prepared through overexpression in *E*. *coli*. The measured molecular weight of 6×His-[C95S]FGF2 by mass spectrometry was consistent with its theoretical value (measured value 18781.0; theoretical value 18780.1), although it seemed slightly larger on SDS-PAGE, likely due to the presence of the N-terminal tag. The biological activity of the recombinant FGF2s was measured as receptor activation potency monitored using a serum response element (SRE)-controlled NanoLuc reporter in HEK293T cells expressing endogenous FGF receptor. As shown Fig 2C, both 6×His-FGF2 and 6×His-[C95S]FGF2 could efficiently activate the SRE pathway, with the calculated EC_50_ values of 58 ± 6 pM (*n* = 3) for 6×His-FGF2 and 100 ± 11 pM (*n* = 3) for 6×His-[C95S]FGF2, consistent with previously reported values of recombinant FGF2 [33,34]. Thus, the N-terminal tag had no detrimental effects on FGF2 activity and we did not remove it in subsequent experiments. In contrast, the mutant FGF2s carrying a Tyr to Ala mutation showed significantly higher EC_50_ values: 309 ± 34 pM (*n* = 3) for 6×His-[Y32A]FGF2 and 794 ± 102 pM (*n* = 3) for 6×His-[Y111A]FGF2. Thus, the receptor activation potency of FGF2 was significantly decreased by both mutations: ~5-fold decrease by Y32A mutation and ~14-fold decrease by Y111A mutation. These results suggested that both Tyr residues are important for FGF2 to activate the endogenous FGF receptor in HEK293T cells, consistent with the fact that they interact with FGF receptor [23–25].

### Chemical conjugation of 6×His-[C95S]FGF2 with the NanoLuc reporter

To prepare the NanoLuc-conjugated FGF2, we used a previously designed 6×His-NanoLuc-Cys that carries a unique exposed Cys residue at the C-terminus for site-specific conjugation with various proteins or peptides [3]. After the unique exposed Cys of 6×His-NanoLuc-Cys was activated by 2,2’-dipyridyl disulfide, the resultant active disulfide bond would react with the unique exposed C77 of 6×His-[C95S]FGF2 and form a disulfide linkage between them. Unfortunately, precipitate always formed after they were mixed together, probably due to the low solubility of the NanoLuc-conjugated FGF2 (FGF2-Luc) in the neutral conjugation solution since its isoelectric point (pI) was around neutral. FGF2 binds the negatively charged heparin; thus, the bound heparin would lower the pI value of the FGF2-Luc conjugate and would increase its solubility in the neutral conjugation solution. As expected, when low molecular weight heparin was added into the conjugation solution, precipitation was inhibited. However, purification of the FGF2-Luc conjugate was difficult, although we tried gel filtration and ion-exchange chromatography. Finally, we designed an on-resin conjugation procedure. First, the purified 6×His-[C95S]FGF2 was bound to the heparin-Sepharose resin and reacted with an excess amount of the activated 6×His-NanoLuc-Cys. Second, the unconjugated 6×His-NanoLuc-Cys was removed by washing and the resin bound FGF2-Luc was eluted with high concentration of heparin. As determined by non-reducing SDS-PAGE (Fig 2D), the washed fraction contained the unreacted 6×His-NanoLuc-Cys (23.0 kDa) and the eluted fraction primarily contained a single band (indicated by an asterisk) with the apparent molecular weight of ~42 kDa, consistent with the theoretical value (41.9 kDa) of the expected FGF2-Luc conjugate. Thus, NanoLuc-conjugated FGF2 could be conveniently prepared using the on-resin conjugation approach. Bioluminescence measurement demonstrated that the FGF2-Luc conjugate retained full NanoLuc activity, with a specific activity of ~1.5 × 10^5^ RLU/fmol when measured on a Spetramax M5 plate reader using a white opaque 96-well plate. Thus, conjugation of a FGF2 moiety was not detrimental to NanoLuc activity.

### Binding of FGF2-Luc to the overexpressed FGFR1

**Fig 3:**
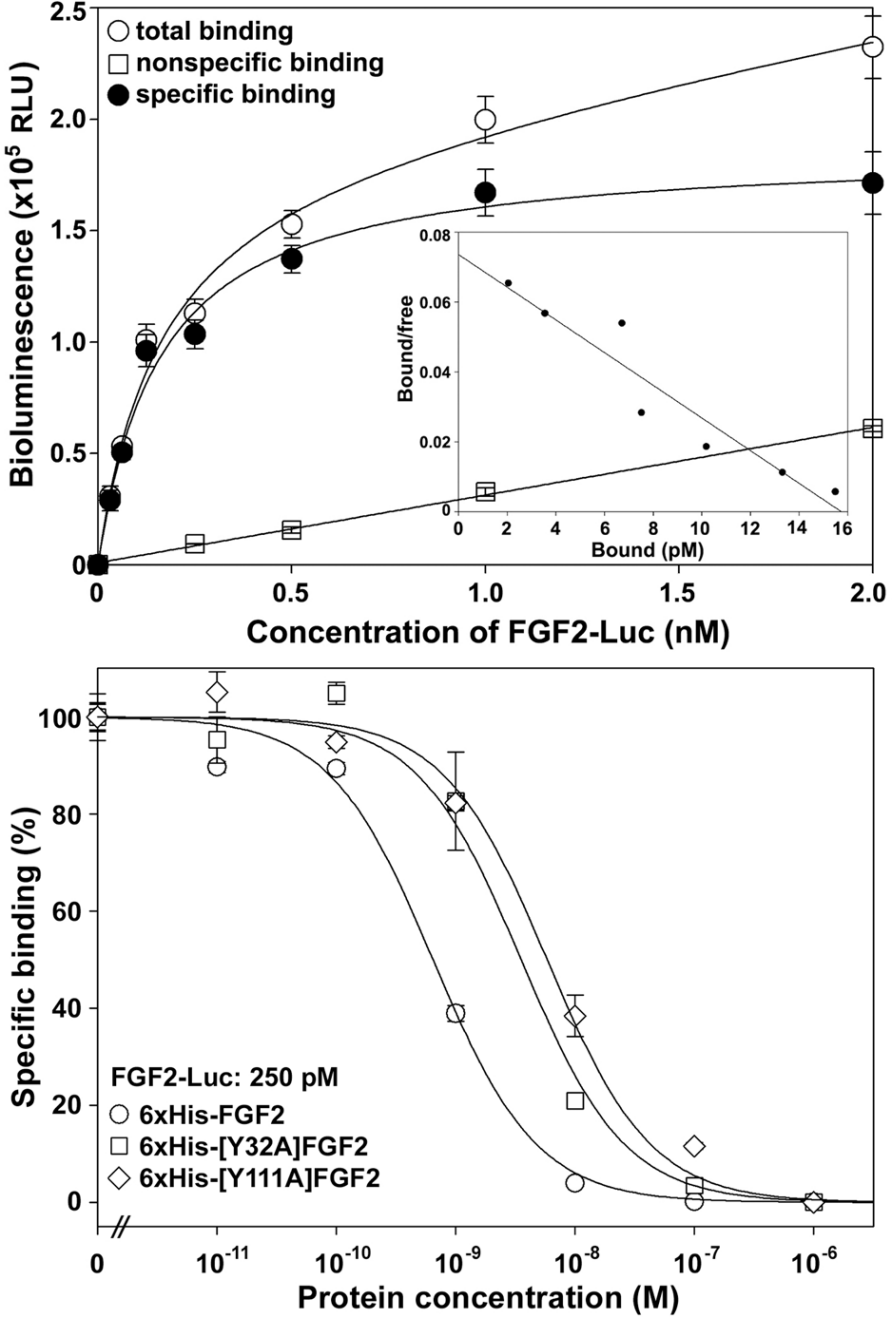
Binding of FGF2-Luc with the overexpressed FGFR1. Living HEK293T cells transiently overexpressing human FGFR1 (transcript variant 2) were used as the receptor source. **(A)** Saturation binding of FGF2-Luc with the overexpressed FGFR1. Nonspecific binding data were obtained by competition with 250 nM of 6×His-FGF2. The measured bioluminescence data were expressed as mean ± SE (*n* = 3). The total binding data were fitted to Y = B_max_X/(K_d_ + X) + N_s_X, specific binding data to Y = B_max_X/(K_d_ + X), and nonspecific binding data to a linear curve. **Inner panel,** Scatchard plot of the specific binding data. **(B)** Competition binding of wild-type or mutant FGF2s with the overexpressed FGFR1 using FGF2-Luc as a tracer. The measured bioluminescence data were expressed as mean ± SE (*n* = 3) and fitted with sigmoidal curves using the SigmaPlot10.0 software.

To test whether the FGF2-Luc conjugate retained receptor-binding affinity, we performed a saturation binding assay using the overexpressed human FGFR1 (transcript variant 2) as a receptor source. As shown in Fig 3A, FGF2-Luc bound living HEK293T cells overexpressing FGFR1 in a typical saturation manner, with a calculated dissociation constant (K_d_) of 161 ± 21 pM (*n* = 3) that was consistent with the previously measured K_d_ values of the radioactive ^125^I-labeled FGF2 tracers [26–32]. Thus, FGF2-Luc retained high binding affinity with the overexpressed receptor FGFR1, suggesting that the conjugated NanoLuc moiety did not negatively affect FGF2 receptor-binding because its conjugation site is far from the receptor-binding site. The calculated maximal binding capacity (B_max_) was 186000 ± 7000 RLU per ~4 × 10^4^ cells, equal to ~19000 receptors/cell calculated according to FGF2-Luc specific activity of 1.5 × 10^5^ RLU/fmol. In contrast, NanoLuc itself had no detectable specific binding with these cells, suggesting that the measured specific binding of FGF2-Luc was from the conjugated FGF2 moiety. The Scatchard plot of the specific binding data was a linear curve (Fig 3A, inner panel), confirming one-site binding of FGF-Luc with the overexpressed FGFR1. As shown in Fig 3B, when FGF2-Luc was used as a tracer, competition binding of 6×His-FGF2 with the tracer on the overexpressed FGFR1 was a typical sigmoidal curve, with a calculated IC_50_ value of 0.65 ± 0.06 nM (*n* = 3). For the mutant FGF2s carrying a Try to Ala mutation, they showed significantly higher IC_50_ values compared to wild-type 6×His-FGF2 (Fig 3B): 3.45 ± 0.59 nM (*n* = 3) for 6×His-[Y32A]FGF2 and 5.88 ± 0.99 nM (*n* = 3) for 6×His-[Y111A]FGF2. Thus, both mutations significantly decreased the binding potency of FGF2 with the overexpressed FGFR1, ~5-fold decrease by Y32A mutation and ~9-fold decrease by Y111A mutation. Thus, FGF2-Luc could monitor binding of various ligands with the overexpressed receptor FGFR1, representing a novel non-radioactive tracer for ligand–receptor interaction studies.

### Binding of FGF2-Luc to the endogenous FGF receptor

**Fig 4:**
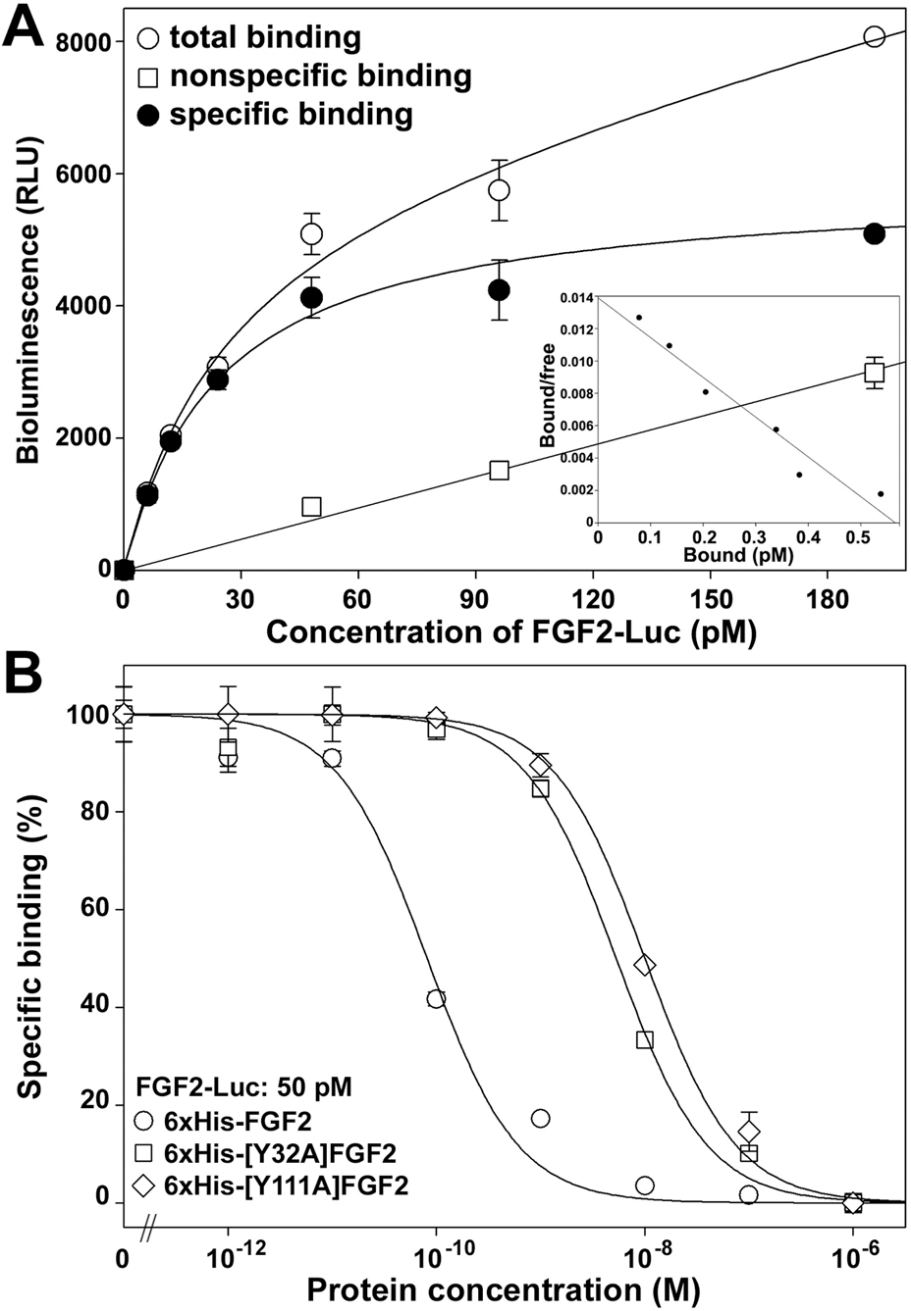
Binding of FGF2-Luc with the endogenous FGF receptor. Untransfected HEK293T cells were used as the receptor source. **(A)** Saturation binding of FGF2-Luc with the endogenous FGF receptor. Nonspecific binding data were obtained by competition with 250 nM of 6×His-FGF2. The measured bioluminescence data were expressed as mean ± SE (*n* = 3). The total binding data were fitted to Y = B_max_X/(K_d_ + X) + N_s_X, specific binding data to Y = B_max_X/(K_d_ + X), and nonspecific binding data to a linear curve. **Inner panel,** Scatchard plot of the specific binding data. **(B)** Competition binding of wild-type or mutant FGF2s with the endogenous FGF receptor using FGF2-Luc as a tracer. The measured bioluminescence data were expressed as mean ± SE (*n* = 3) and fitted with sigmoidal curves using the SigmaPlot10.0 software.

To test the sensitivity of the novel bioluminescent FGF2 tracer, we performed the saturation binding assay using the endogenous FGF receptor. As shown in Fig 4A, FGF2-Luc bound the untransfected HEK293T cells in a typical saturation manner, with a calculated K_d_ value of 25 ± 4 pM (*n* = 3) and a B_max_ value of 5850 ± 240 RLU per ~5 × 10^4^ cells, equal to ~470 receptors/cell. Thus, the bioluminescent FGF2 tracer was sensitive enough to detect the endogenous FGF receptor. The Scatchard plot of the specific binding data was a linear curve (Fig 4A, inner panel), suggesting that HEK293T cells primarily expressed one kind of high affinity FGF receptor. When FGF2-Luc was used as a tracer in competition binding assays using the untransfected HEK293T cells as receptor source (Fig 4B), typical sigmoidal curves were obtained for wild-type or mutant FGF2s, but they had significantly different IC_50_ values: 81 ± 9 pM (*n* = 3) for wild-type 6×His-FGF2, 5.4 ± 0.6 nM (*n* = 3) for 6×His-[Y32A]FGF2, and 10.0 ± 1.1 nM (*n* = 3) for 6×His-[Y111A]FGF2. Thus, both mutant FGF2s showed approximately 100-fold lower binding potency than wild-type FGF2 towards the endogenous FGF receptor, suggesting that both Tyr residues are critical for binding of the endogenous FGF receptor. In summary, the novel bioluminescent FGF2 tracer represents an ultrasensitive non-radioactive tracer for binding assays of FGF receptors with various ligands.

### Application of the novel bioluminescent binding assay to other protein/peptide hormones

In our previous and present studies, we validated the novel bioluminescent binding assay using a variety of protein/peptide hormones with different size and distinct receptors, including relaxin (receptor RXFP1) [1], INSL3 (receptor RXFP2) [5], chimeric relaxin family peptide R3/I5 (receptor RXFP3 and RXFP4) [35], ghrelin (receptor GSHR1a) [3], leukemia inhibitory factor (receptor LIFR/gp130) [4], erythropoietin (receptor EPOR) [2], and FGF2 (receptor FGFR1–4). Although the NanoLuc reporter is large, its attachment did not negatively affect the receptor-binding of a wide range of protein/peptide hormones, provided it was attached to an appropriate position using an appropriate linkeFor wide application of the novel binding assay, we developed two methods for convenient preparation of the bioluminescent ligands, that is, the chemical conjugation method and the genetic fusion method [7]. For the chemical conjugation method, an engineered NanoLuc reporter carrying a unique reactive moiety was chemically conjugated with a rationally designed recombinant or synthetic protein/peptide that also carries a unique reactive moiety at an appropriate position. For the genetic fusion method, the NanoLuc reporter was fused at the N-terminus or C-terminus of the target protein/peptide through a suitable peptide linker and the resultant NanoLuc-fused protein was overexpressed in suitable host cells. In future studies, these methods can be applied to other protein/peptide hormones for convenient preparation of the NanoLuc-based tracers for novel bioluminescent ligand–receptor binding assays.

## Supporting Information

S1 Fig The nucleotide sequence and amino acid sequence of the recombinant 6×His-FGF2. The N-terminal 6×His-tag and enterokinase cleavage site (DDDDK) were underlined. The nucleotide cleavage sites of restriction enzymes NdeI and EcoRI were shaded. The exposed cysteine residue (C95) replaced by serine in 6×His-[C95S]FGF2 was shown in red. The exposed Cys residue (C77) used for conjugation with the engineered NanoLuc reporter was shown in blue. The Tyr residues (Y32 and Y111) mutated in 6×His-[Y32A]FGF2 and 6×His-[Y111A]FGF2 were shown in green.
(DOC)

## References

[1] Wu QP, Zhang L, Shao XX, Wang JH, Gao Y, Liu YL, et al Application of the novel bioluminescent ligand−receptor binding assay to relaxin-RXFP1 system for interaction studies. Amino Acids 2016; 48: 1099–1107. 10.1007/s00726-015-2146-3 26767372

[2] Song G, Wu QP, Xu T, Liu YL, Xu ZG, Zhang SF, et al Quick preparation of nanoluciferase-based tracers for novel bioluminescent receptor-binding assays of protein hormones: using erythropoietin as a model. J Photochem Photobiol B 2015; 153: 311–316. 10.1016/j.jphotobiol.2015.10.014 26506452

[3] Liu Y, Shao XX, Zhang L, Song G, Liu YL, Xu ZG, et al Novel bioluminescent receptor-binding assays for peptide hormones: using ghrelin as a model. Amino Acids 2015; 47: 2237–2243. 10.1007/s00726-015-2009-y 26002812

[4] He SX, Song G, Shi JP, Guo YQ, Guo ZY. Nanoluciferase as a novel quantitative protein fusion tag: Application for overexpression and bioluminescent receptor-binding assays of human leukemia inhibitory factor. Biochimie 2014; 106: 140–148. 10.1016/j.biochi.2014.08.012 25179300

[5] Zhang L, Song G, Xu T, Wu QP, Shao XX, Liu YL, et al A novel ultrasensitive bioluminescent receptor-binding assay of INSL3 through chemical conjugation with nanoluciferase. Biochimie 2013; 95: 2454–2459. 10.1016/j.biochi.2013.09.008 24056075

[6] Hall MP, Unch J, Binkowski BF, Valley MP, Butler BL, Wood MG, et al Engineered luciferase reporter from a deep sea shrimp utilizing a novel imidazopyrazinone substrate. ACS Chem Biol 2012; 7: 1848–1857. 10.1021/cb3002478 22894855 PMC3501149

[7] Liu YL, Guo ZY. Novel bioluminescent binding assays for interaction studies of protein/peptide hormones with their receptors. Amino Acids 2016; 48: 1151–1160. 10.1007/s00726-016-2220-5 27020777

[8] Itoh N, Ohta H, Konishi M. Endocrine FGFs: Evolution, Physiology, Pathophysiology, and Pharmacotherapy. Front Endocrinol (Lausanne) 2015; 6: 154. 26483756 10.3389/fendo.2015.00154 PMC4586497

[9] Fernandes-Freitas I, Owen BM. Metabolic roles of endocrine fibroblast growth factors. Curr Opin Pharmacol 2015; 25: 30–35. 10.1016/j.coph.2015.09.014 26531325

[10] Powers CJ, McLeskey SW, Wellstein A. Fibroblast growth factors, their receptors and signaling. Endocr Relat Cancer 2000; 7: 165–197. 11021964 10.1677/erc.0.0070165

[11] Szebenyi G, Fallon JF. Fibroblast growth factors as multifunctional signaling factors. Int Rev Cytol 1999; 185: 45–106. 9750265 10.1016/s0074-7696(08)60149-7

[12] Katoh M, Nakagama H. FGF receptors: cancer biology and therapeutics. Med Res Rev 2014; 34: 280–300. 10.1002/med.21288 23696246

[13] Goetz R, Mohammadi M. Exploring mechanisms of FGF signalling through the lens of structural biology. Nat Rev Mol Cell Biol 2013; 14: 166–180. 10.1038/nrm3528 23403721 PMC3695728

[14] Turner N, Grose R. Fibroblast growth factor signalling: from development to cancer. Nat Rev Cancer 2010; 10: 116–129. 10.1038/nrc2780 20094046

[15] Armelin HA. Pituitary extracts and steroid hormones in the control of 3T3 cell growth. Proc Natl Acad Sci USA 1973; 70: 2702–2706. 4354860 10.1073/pnas.70.9.2702 PMC427087

[16] Gospodarowicz D. Localisation of a fibroblast growth factor and its effect alone and with hydrocortisone on 3T3 cell growth. Nature 1974; 249: 123–127. 4364816 10.1038/249123a0

[17] Abraham JA, Mergia A, Whang JL, Tumolo A, Friedman J, Hjerrild KA, et al Nucleotide sequence of a bovine clone encoding the angiogenic protein, basic fibroblast growth factor. Science 1986; 233: 545–548. 2425435 10.1126/science.2425435

[18] Kurokawa T, Sasada R, Iwane M, Igarashi K. Cloning and expression of cDNA encoding human basic fibroblast growth factor. FEBS Lett 1987; 213: 189–194. 2435575 10.1016/0014-5793(87)81489-8

[19] Prats H, Kaghad M, Prats AC, Klagsbrun M, Lélias JM, Liauzun P, et al High molecular mass forms of basic fibroblast growth factor are initiated by alternative CUG codons. Proc Natl Acad Sci USA 1989; 86: 1836–1840. 2538817 10.1073/pnas.86.6.1836 PMC286799

[20] La Venuta G, Zeitler M, Steringer JP, Müller HM, Nickel W. The startling properties of fibroblast growth factor 2: how to exit mammalian cells without a signal peptide at hand. J Biol Chem 2015; 290: 27015–27020. 10.1074/jbc.R115.689257 26416892 PMC4646381

[21] Zhu X, Komiya H, Chirino A, Faham S, Fox GM, Arakawa T, et al Three-dimensional structures of acidic and basic fibroblast growth factors. Science 1991; 251: 90–93. 1702556 10.1126/science.1702556

[22] Ago H, Kitagawa Y, Fujishima A, Matsuura Y, Katsube Y. Crystal structure of basic fibroblast growth factor at 1.6 A resolution. J Biochem 1991; 110: 360–363. 1769963 10.1093/oxfordjournals.jbchem.a123586

[23] Plotnikov AN, Hubbard SR, Schlessinger J, Mohammadi M. Crystal structures of two FGF-FGFR complexes reveal the determinants of ligand-receptor specificity. Cell 2000; 101: 413–424. 10830168 10.1016/s0092-8674(00)80851-x

[24] Schlessinger J, Plotnikov AN, Ibrahimi OA, Eliseenkova AV, Yeh BK, Yayon A, et al Crystal structure of a ternary FGF-FGFR-heparin complex reveals a dual role for heparin in FGFR binding and dimerization. Mol Cell 2000; 6: 743–750. 11030354 10.1016/s1097-2765(00)00073-3

[25] Plotnikov AN, Schlessinger J, Hubbard SR, Mohammadi M. Structural basis for FGF receptor dimerization and activation. Cell 1999; 98: 641–650. 10490103 10.1016/s0092-8674(00)80051-3

[26] Neufeld G, Gospodarowicz D. The identification and partial characterization of the fibroblast growth factor receptor of baby hamster kidney cells. J Biol Chem 1985; 260: 13860–13868. 2997183

[27] Moscatelli D. High and low affinity binding sites for basic fibroblast growth factor on cultured cells: absence of a role for low affinity binding in the stimulation of plasminogen activator production by bovine capillary endothelial cells. J Cell Physiol 1987; 131: 123–130. 3032990 10.1002/jcp.1041310118

[28] Sharma A, Dahiya R. Bovine basic fibroblast growth factor: identification and binding of its receptor on PC12 cells. Biomed Biochim Acta 1988; 47: 975–983. 2855399

[29] Olwin BB, Hauschka SD. Identification of the fibroblast growth factor receptor of Swiss 3T3 cells and mouse skeletal muscle myoblasts. Biochemistry 1986; 25: 3487–3492. 3013291 10.1021/bi00360a001

[30] Olwin BB, Hauschka SD. Cell type and tissue distribution of the fibroblast growth factor receptor. J Cell Biochem 1989; 39: 443–454. 2542345 10.1002/jcb.240390410

[31] Dionne CA, Crumley G, Bellot F, Kaplow JM, Searfoss G, Ruta M, et al Cloning and expression of two distinct high-affinity receptors cross-reacting with acidic and basic fibroblast growth factors. EMBO J 1990; 9: 2685–2692. 1697263 10.1002/j.1460-2075.1990.tb07454.x PMC551973

[32] Ornitz DM, Leder P. Ligand specificity and heparin dependence of fibroblast growth factor receptors 1 and 3. J Biol Chem 1992; 267: 16305–16311. 1379594

[33] Squires CH, Childs J, Eisenberg SP, Polverini PJ, Sommer A. Production and characterization of human basic fibroblast growth factor from *Escherichia coli*. J Biol Chem 1988; 263: 16297–16302. 3053689

[34] Kroiher M, Raffioni S, Steele RE. Single step purification of biologically active recombinant rat basic fibroblast growth factor by immobilized metal affinity chromatography. Biochim Biophys Acta 1995; 1250: 29–34. 7612650 10.1016/0167-4838(95)00060-8

[35] Hu MJ, Shao XX, Wang JH, Wei D, Liu YL, Xu ZG, et al Identification of hydrophobic interactions between relaxin-3 and its receptor RXFP3: implication for a conformational change in the B-chain C-terminus during receptor binding. Amino Acids. Forthcoming 2016, 10.1007/s00726-016-2260-x 27193232

