# Supplementary material for: Novel Bioluminescent Binding Assays for Ligand–Receptor Interaction Studies of the Fibroblast Growth Factor Family
Source: PLoS One. 2016 Jul 14;11(7):e0159263. doi: 10.1371/journal.pone.0159263 (PMC4944982; doi:10.1371/journal.pone.0159263)
Supplement: S1 Fig — The N-terminal 6×His-tag and enterokinase cleavage site (DDDDK) were underlined. The nucleotide cleavage sites of restriction enzymes NdeI and EcoRI were shaded. The exposed cysteine residue (C95) replaced by serine in 6×His-[C95S]FGF2 was shown in red. The exposed Cys residue (C77) used for conjugation with the engineered NanoLuc reporter was shown in blue. The Tyr residues (Y32 and Y111) mutated in 6×His-[Y32A]FGF2 and 6×His-[Y111A]FGF2 were shown in green. (DOC) [file pone.0159263.s001.doc]

1 ATG CAT CAC CAT CAC CAC CAT ATG GAT GAC GAT GAC AAA GCG GCC GGG AGC ATC ACC ACG CTG CCG GCA TTG CCG

TAC GTA GTG GTA GTG GTG GTA TAC CTA CTG CTA CTG TTT CGC CGG CCC TCG TAG TGG TGC GAC GGC CGT AAC GGC

M H H H H H H M D D D D K A A G S I T T L P A L P

76 GAA GAT GGC GGC TCC GGT GCC TTC CCG CCA GGC CAT TTT AAA GAC CCG AAA CGT CTG TAT TGC AAA AAC GGT GGC

CTT CTA CCG CCG AGG CCA CGG AAG GGC GGT CCG GTA AAA TTT CTG GGC TTT GCA GAC ATA ACG TTT TTG CCA CCG

E D G G S G A F P P G H F K D P K R L **Y** C K N G G

151 TTC TTT CTG CGC ATT CAC CCA GAT GGC CGT GTT GAC GGT GTC CGC GAG AAG AGC GAT CCT CAT ATC AAA CTG CAG

AAG AAA GAC GCG TAA GTG GGT CTA CCG GCA CAA CTG CCA CAG GCG CTC TTC TCG CTA GGA GTA TAG TTT GAC GTC

F F L R I H P D G R V D G V R E K S D P H I K L Q

226 CTT CAA GCG GAA GAG CGT GGT GTT GTG TCT ATT AAA GGG GTG TGT GCT AAC CGT TAT CTC GCG ATG AAG GAA GAT

GAA GTT CGC CTT CTC GCA CCA CAA CAC AGA TAA TTT CCC CAC ACA CGA TTG GCA ATA GAG CGC TAC TTC CTT CTA

L Q A E E R G V V S I K G V **C** A N R Y L A M K E D

301 GGT CGC TTA CTG GCC TCA AAA TGT GTA ACC GAC GAG TGC TTC TTT TTC GAA CGT CTG GAA AGC AAT AAC TAC AAT

CCA GCG AAT GAC CGG AGT TTT ACA CAT TGG CTG CTC ACG AAG AAA AAG CTT GCA GAC CTT TCG TTA TTG ATG TTA

G R L L A S K **C** V T D E C F F F E R L E S N N **Y** N

376 ACT TAC CGC TCG CGT AAA TAT ACC AGT TGG TAT GTG GCA CTG AAA CGC ACG GGG CAG TAC AAA CTG GGT TCC AAA

TGA ATG GCG AGC GCA TTT ATA TGG TCA ACC ATA CAC CGT GAC TTT GCG TGC CCC GTC ATG TTT GAC CCA AGG TTT

T Y R S R K Y T S W Y V A L K R T G Q Y K L G S K

451 ACC GGC CCG GGC CAG AAA GCT ATT CTG TTT CTG CCC ATG TCT GCG AAG AGC TAA GAA TTC

TGG CCG GGC CCG GTC TTT CGA TAA GAC AAA GAC GGG TAC AGA CGC TTC TCG ATT CTT AAG

T G P G Q K A I L F L P M S A K S *
